# Vegetable Soups in the Treatment of Children's Anæmias

**Published:** 1914-04-11

**Authors:** 


### Vegetable Soups in the Treatment of Children's Anæmias.

Some years ago the Munich School pointed out the advantage of vegetable broths in the treatment of children, especially young infants, suffering from malnutrition due to intestinal causes. Quite young infants, suffering from diarrhœa with great emaciation and tetany, fed on such broths were found to recover, to put on flesh, and to improve rapidly even in conditions where other methods of treatment had proved failures. Since then the treatment has been tried in many other conditions, generally with excellent results, but it is not well known in this country.

Vegetable broths are particularly useful for children suffering from malnutrition, anæmia, gastric atony, asthenia, spasmophilia, and that rather prevalent condition which the practitioner vaguely styles "nervous debility." They cannot wholly take the place of the regular tonics, such as iron, the formates and phosphates, the stomach bitters, and nerve stimulants, but they serve as adjuncts to these, and as such are very useful. The only contraindication for their use seems to be actual ulceration of the intestines or tubercular peritonitis.

Of the vegetables commonly used in the preparation of these broths, carrots, artichokes, and parsnips are undoubtedly the best, but other kinds may be used. Beetroot prepared as a "bortsch" and given with cream, for instance, is an excellent broth, although the basis here is generally animal and not vegetable proteid. As an example of the preparation of a broth, we may take carrot soup, which is easy to prepare and which in general yields the best results. A pound of young carrots is taken, and the roots are carefully scraped with a sharp knife or piece of glass. The carrots are then sliced fine and put in a stewpan with some butter and allowed to braize gently over a fire. After forty minutes' slow stewing, during which the pan is frequently shaken so as to prevent burning—the lid being kept on meanwhile—the stewpan is removed from the range, and the contents are finely mashed with a fork or passed through a fine colander. This fine mash is now placed in a casserole, two pints of cold water are added, and the mixture is slowly boiled until it is reduced to the consistency of thin porridge. A few tablespoonfuls of cream are now added, and the mixture made more liquid by the addition of two further pints of cold water, and rapidly brought to the boil, care being taken to prevent the formation of any lumps. When the soup has boiled any scum is carefully removed. If these directions have been carefully carried out, the result is a fairly homogeneous, yellowish broth of the consistency of thin cream, pleasant and satisfying to the taste. A drop of this placed under the low power of the microscope shows thin shreds of vegetable matter, fat droplets, and starch granules floating in a semi-opaque yellowish liquid. The addition of meat extract, stock made from chicken or mutton bones, or any of the proprietary meat jellies or essences, greatly adds to the richness of the broth, but is usually inadvisable if the soup is required for young infants. Carrots contain an appreciable amount of iron, and such a broth so prepared is relatively rich in organic iron compounds, which are easily assimilated. The cellulose acts as a mechanical stimulant of the intestines, and probably improves absorption in the small intestine, while it stimulates peristaltic movement in the colon. Similar soups may be prepared from artichokes, parsnips, turnips, and beets, and it is advisable to ring the changes upon these various vegetables, since children very soon tire of a carrot diet.

The broth is administered to infants either by spoon-feeding or by the bottle. In the latter case it has first to be strained through coarse muslin, so as to eliminate the coarser particles, which may otherwise clog the teat. Older children may either drink it from a feeding-cup or take it like ordinary soup in a plate. The broth must be made freshly each day, but the stock of the first day may be incorporated with that of the new broth if desired. If it is wished to keep the soup for a few hours after being made, it must be poured into a scalded basin and kept covered with wet muslin to protect it from dust and other impurities, and must be boiled again before being used.

